# Results of Beta Secretase-Inhibitor Clinical Trials Support Amyloid Precursor Protein-Independent Generation of Beta Amyloid in Sporadic Alzheimer’s Disease

**DOI:** 10.3390/medsci6020045

**Published:** 2018-06-02

**Authors:** Vladimir Volloch, Sophia Rits

**Affiliations:** 1 Deptartment of Developmental Biology, Harvard School of Dental Medicine, Boston, MA 02115, USA; 2 Howard Hughes Medical Institute at Children’s Hospital, Boston, MA 02115, USA; rits@crystal.harvard.edu; 3 Deptartment of Biological Chemistry and Molecular Pharmacology, Harvard Medical School, Boston, MA 02115, USA

**Keywords:** Alzheimer’s disease, amyloid precursor protein, familial Alzheimer’s disease, sporadic Alzheimer’s disease, β-site amyloid precursor protein-cleaving enzyme 1 inhibitors, amyloid precursor protein-independent generation of β amyloid

## Abstract

The present review analyzes the results of recent clinical trials of β secretase inhibition in sporadic Alzheimer’s disease (SAD), considers the striking dichotomy between successes in tests of β-site Amyloid Precursor Protein-Cleaving Enzyme (BACE) inhibitors in healthy subjects and familial Alzheimer’s disease (FAD) models versus persistent failures of clinical trials and interprets it as a confirmation of key predictions for a mechanism of amyloid precursor protein (APP)-independent, β secretase inhibition-resistant production of β amyloid in SAD, previously proposed by us. In light of this concept, FAD and SAD should be regarded as distinctly different diseases as far as β-amyloid generation mechanisms are concerned, and whereas β secretase inhibition would be neither applicable nor effective in the treatment of SAD, the β-site APP-Cleaving Enzyme (BACE) inhibitor(s) deemed failed in SAD trials could be perfectly suitable for the treatment of FAD. Moreover, targeting the aspects of Alzheimer’s disease (AD) other than cleavages of the APP by β and α secretases should have analogous impacts in both FAD and SAD.

## 1. Introduction

Beta amyloid, Aβ, the peptide associated with and widely believed to have a pivotal early role in the etiology of Alzheimer’s disease (AD), is generated ostensibly by proteolytic cleavages of a much larger molecule, β amyloid precursor protein, βAPP. In the amyloidogenic proteolytic pathway, two sequential cleavages of βAPP are involved in the production of Aβ. The first is a cleavage of βAPP by the β secretase enzyme. It occurs between residues 671 and 672 of the βAPP molecule (isoform 770 numbering), generating the N-terminus of Aβ, yielding the 12 kDa membrane-bound C-terminal fragment, C99 (residues 672–770), releasing a large ectodomain of βAPP, soluble sAPPβ (residues 1–671), and precluding the activity of α secretase that cleaves βAPP within its Aβ segment but cannot cut within C99 or Aβ [1,2,3]. The second cleavage, by γ secretase activity, occurs at one of multiple sites within C99 and generates the C-terminus of Aβ. Thus released, Aβ is secreted from the cell. The size of Aβ ranges from 36 to 43 amino acids, with Aβ40 being the most abundant species normally formed. Studies of the inherited forms of the disease, familial Alzheimer’s disease (FAD), strongly indicated that cerebral Aβ accumulation is essential for and underlies the etiology of the disease [4,5,6]. This notion was formalized in a putative theory of Alzheimer’s disease known as “Amyloid Cascade Hypothesis” (ACH) [7,8,9,10,11,12]. ACH has become the dominant model of AD pathogenesis and has been guiding the development of potential treatments; most therapeutic strategies attempted to date have been based on this model. Several alternative theories of Alzheimer’s disease AD have also been proposed. They include Mitochondrial Cascade Hypothesis (MCH), (reviewed in ref. [13]), Presenilin Hypothesis (PH), and βAPP Matrix Approach (AMA) (both reviewed in ref. [14]). However, since all preclinical tests and clinical trials discussed below have been designed within the framework of ACH, the present review is limited to this framework. Over two hundred autosomal dominant mutations associated with FAD have been identified in genes for βAPP and presenilins, which are the components of γ secretase complex [6]. In βAPP gene most of the mutations cluster around secretases cleavage sites and increase either the production of total Aβ or the relative proportion of a more neurotoxic 42-residue form of Aβ, Aβ42. In terms of the ACH, there is little doubt that the abnormal processing of βAPP and increased production of total Aβ or its 42-amino acid isoform are pivotal events in the pathogenesis of FAD. Although the number of individuals affected by FAD is substantial, in relative terms this form of the disease is quite rare, representing less than 5% (less than 1% by some estimates) of the total Alzheimer’s disease burden [5,14,15]. Since the pathological lesions and symptoms in the non-hereditary form of the disease, sporadic Alzheimer’s disease (SAD), are analogous to those seen in the familial forms, it has been assumed that abnormal amyloidogenic proteolytic processing of βAPP also underlies the pathogenesis of SAD [4,5].

## 2. Success of β-site Amyloid Precursor Protein-Cleaving Enzyme Inhibitors in Preclinical Tests 

In the framework of ACH, it was understood early on that the inhibition of Aβ production might benefit affected individuals. In light of the above discussion, β secretase activity was viewed, due in part to its temporal position at the top of the cascade sequence, as a strategic target of choice. Therefore, since the identification of BACE (β-site APP-Cleaving Enzyme) as β secretase [16,17,18], it has become the primary therapeutic target for treatment of AD. Designing BACE-inhibiting agents presented major challenges of cell penetration, oral bioavailability, metabolic clearance, and brain access. Intense efforts, mainly by the pharmaceutical industry, led to the development of a number of brain penetrant small molecule BACE inhibitors that have been vigorously investigated. The results obtained in the early investigations of BACE inhibition, first appearing around 2007 [19,20,21,22,23,24,25,26,27], are truly striking. As an example, Merck researchers reported in 2012 the discovery of “compound 16”, which robustly reduced cortex and cerebrospinal fluid (CSF) levels of Aβ when administered orally to rats [28]. Continuous efforts to improve upon “compound 16” culminated in the development of verubecestat (MK-8931). Preclinical tests of this agent achieved dramatic results [29]. Levels of Aβ and sAPPβ were reduced by up to 90% in plasma, brain, and CSF after even a single administration of verubecestat to healthy subjects including rats, monkeys, and humans [29]. The acute reduction of over 80% in CSF and cortical Aβ and sAPPβ produced by verubecestat was maintained after chronic administration for nine months in monkeys [29]. Because of its favorable initial safety profile and its ability to markedly reduce cerebral and CSF Aβ and sAPPβ concentrations, verubecestat was the first BACE inhibitor to progress to phase III clinical trials. Preclinical evaluation of a number of independently developed BACE inhibitors, such as BI-1181181, LY-2811376, LY-2886721, AZD-3293 (lanabacestat, LY-3314814), CNP-520, E-2609 (elenbacestat), JNJ-54861911, CTS-21166, HPP-854, PF-05297909, RG-7129, TAK-070, VTP-37948 yielded similarly impressive results in animals and healthy volunteers and all these agents have entered clinical trials.

## 3. Inhibition of Beta Secretase Activity Rescues Functional Impairments in Animal Models of Familial Alzheimer’s Disease

With the ability to significantly reduce the production and lower the levels of Aβ thus established, the question remained whether such a reduction would translate into a “treatment” of the disease. This question was answered resolutely and convincingly, at least in the animal models of FAD, in two recent studies using different approaches to inhibit β secretase activity. One study utilized BACE inhibitor NB-360 [30]. It was based on a previous study [31] showing NB-360 to be a potent, brain penetrable BACE inhibitor capable of completely blocking Aβ deposition in the brains of βAPP transgenic mice, as well as of rats and dogs. Moreover, this inhibitor blocked the accumulation of activated inflammatory cells in the brains of βAPP transgenic mice. The more recent study with NB-360 [30] further assessed the notion that suppression of Aβ production can have beneficial downstream effects on the progression of Alzheimer’s disease. Using histochemistry, in vivo Ca^2+^ imaging, and behavioral analyses in a mouse model of FAD, the authors demonstrated that along with reducing prefibrillary Aβ surrounding plaques, the inhibition of BACE activity rescued neuronal hyperactivity, impaired long-range circuit function, and memory defects. That all these effects were due to the inhibition of Aβ production was strongly indicated by the observation that functional neuronal impairments reappeared after the infusion of soluble Aβ [30]. 

In the second study [32], mimicking BACE1 inhibition in adults, the authors generated BACE1 conditional knockout (BACE1 ^fl/fl^) mice and bred them with ubiquitin-Cre mice to induce the deletion of BACE1 after passing early developmental stages. Strikingly, sequential and increased deletions of BACE1 in an adult FAD mouse model were capable of reversing amyloid deposition and resulted in a significant improvement in gliosis and neuritic dystrophy. Moreover, in correlation with amyloid plaque reversal, it also significantly improved synaptic functions, as was determined by long-term potentiation and contextual fear conditioning experiments. These studies offered great hope that the sustained inhibition of BACE1 activity can constitute a treatment, or at least be beneficial, for AD patients. This assumption was tested in several clinical trials.

## 4. BACE Inhibitors Are Completely Inefficient in Treatment of Sporadic Alzheimer’s Disease

The results of clinical trials of BACE inhibitors, however, do not support this hope in the case of sporadic AD. All BACE inhibitor clinical trials that ended to date, ended in failure. Some trials, such as that of BI-1181181, LY-2811376, LY-2886721 and RG-7129, were terminated because of technical and safety issues. On the other hand, there were no such issues in the trials of the verubecestat (MK-8931). This agent was shown to be very efficient in suppressing Aβ production in preclinical tests and was proven safe in clinical trials. Yet, its Phase III, 2000 patient-strong Enhanced Peri-Operative Care for High-risk patients (“EPOCH”) trial in mild to moderate SAD patients was terminated prematurely in February 2017 for the lack of efficacy, with an interim analysis by an external data-monitoring committee giving the trial “virtually no chance of finding a positive effect”. At that time, a separate large Phase III clinical trial of verubecestat in prodromal SAD patients, the β Amyloid Production and Effects on Cognition Study (“APECS”), set to run until 2019, was continued as investigators found no signs of safety issues. In February 2018, however, this trial, too, was terminated prematurely and for the same reason: lack of efficacy. The clinical trials of several other BACE inhibitors are still in progress but the verubecestat results do not inspire confidence in their successful outcome.

Why such a dichotomy between highly successful BACE inhibition-mediated treatments of the disease in mouse models versus the failure of the same approach, with an efficient and safe agent, in human clinical trials? One possible explanation for such a discrepancy is a physiological difference between mice and humans. Whereas this could be a contributing factor, the main reason may be fundamentally different. To explain the dramatic discordance between the outcomes of animal studies and of human trials, we would like to advance the notion that two etiologically and mechanistically different, yet symptomatically similar, if not identical, diseases were treated in mouse studies versus human trials. Mouse AD models imitate, by design, FAD. In all such models, multiple copies of human βAPP gene or its fragment containing β amyloid coding sequences, usually carrying one or more of known AD mutations, are inserted into the genome under the control of a strong promoter. Undisputedly, in FAD, as well as in all of its mouse models and healthy subjects used in BACE inhibitor studies, Aβ is generated solely by the proteolytic cleavage of βAPP by β- and γ-secretases. Even if mouse models were constructed by insertion of a large number of wild-type βAPP genes, this would still be the case; such a mouse would not be a model of sporadic AD. BACE inhibitors should be effective in such a setting and they are. Moreover, for the same reasons, BACE inhibitors should be effective in FAD patients. However, all clinical trials of BACE inhibitors were and are either strictly or for all practical purposes those of sporadic AD. This is because FAD cases constitute less than 5% of the AD burden [5,14,15]. Therefore, even if mixed FAD/SAD cohorts were used in a trial, assuming that their proportion reflects the natural frequency of familial AD cases, FAD patients would constitute no more than 5% of a cohort and would have little impact on the outcomes of a trial. 

## 5. Results of Clinical Trials Can Be Explained by APP-Independent and BACE Inhibition-Insensitive Generation of Beta Amyloid in Sporadic Alzheimer’s Disease

One rational explanation for the dichotomy referred to above is that in the majority of sporadic AD cases, in addition to conventional βAPP/β secretase-dependent component of Aβ production that operates in FAD (and in healthy subjects), there is another, unconventional, Aβ-generating component in operation, possibly facilitated or enabled by epigenetic changes associated with the disease [33], which is both βAPP- and β secretase-independent. In these (sporadic) cases, administration of safe and effective BACE inhibitors would suppress the βAPP-dependent component, but would have no effects on the second, βAPP- and β secretase-independent, component. The extent of suppression of total Aβ production by BACE inhibitors would depend on the relative input of two components in the generation of Aβ, and if the input of the second significantly exceeds that of the first component, BACE inhibitors would be ineffective both in lowering Aβ levels and in the treatment of SAD. 

What could this second component be? Conceivably, several molecular mechanisms might form a basis for such a process; for example, a shift of the transcription start site to a position that generates short mRNA encoding polypeptides that do not require β secretase cleavage for the generation of Aβ, or an internal initiation of translation of conventional βAPP mRNA that achieves the same result. In fact, a specific mechanism for βAPP- and β secretase-independent production of C99 and, subsequently, Aβ in sporadic AD was previously proposed [34,35,36]. Its central and defined prediction is just this: inhibition of β secretase activity should be highly effective in suppressing Aβ production in FAD but would have little effect on Aβ generation in SAD.

## 6. Generation of Severely 5′-Truncated βAPP mRNA Encoding C99 Fragment of β Amyloid Precursor Protein in Sporadic Alzheimer’s Disease

The proposed mechanism for APP-independent overproduction of Aβ in sporadic AD [34,35,36] is based upon two foundations. The first one is an intriguing possibility suggested by the primary structure of the human βAPP mRNA. In βAPP mRNA, the Aβ-coding segment is preceded immediately and in-frame by the AUG codon normally encoding methionine in position 671 of the APP (isoform 770 numbering). If translation were initiated at this position, it would produce 12kDa C-terminal APP fragment (C99, after the removal of methionine by the N-terminal methionine aminopeptidase) independently of βAPP. Interestingly, the AUG in question is situated within a nucleotide context optimal for the initiation of translation (an “A” in position −3 and a “G” in position +4 relative to the “A” of the AUG codon). In fact, of the twenty AUG codons encoding methionine residues in the βAPP mRNA, only the AUG encoding Met671, not even Met1, is located within an optimal translation initiation context. Such favorable positioning of the AUG encoding Met671 of APP was the basis for a proposal by Breimer and Denny that in Alzheimer’s disease the C99 APP fragment may be generated independently from βAPP by the internal initiation of translation of the intact βAPP mRNA [37]. Such precursor-independent generation of C99 would be an efficient way to overproduce Aβ. This is because (a) C99 is not susceptible to the α secretase cleavage (1–3), (b) cleavage by γ secretase was shown to be not the rate-limiting step in the production of Aβ (1–3), and (c) Aβ produced from C99 containing or lacking signal sequence was shown to be secreted with similar efficiency (2). The possibility of the internal initiation of translation, proposed by Breimer and Denny [37], has been subsequently ruled out by the elegant experiments of Citron and co-investigators [38]. There is, however, another possibility of utilization of the AUG in question as a translation initiation codon, namely the generation of a severely 5′-truncated βAPP mRNA in which the AUG encoding Met671 in the intact mRNA becomes the first AUG codon.

**Figure 1:**
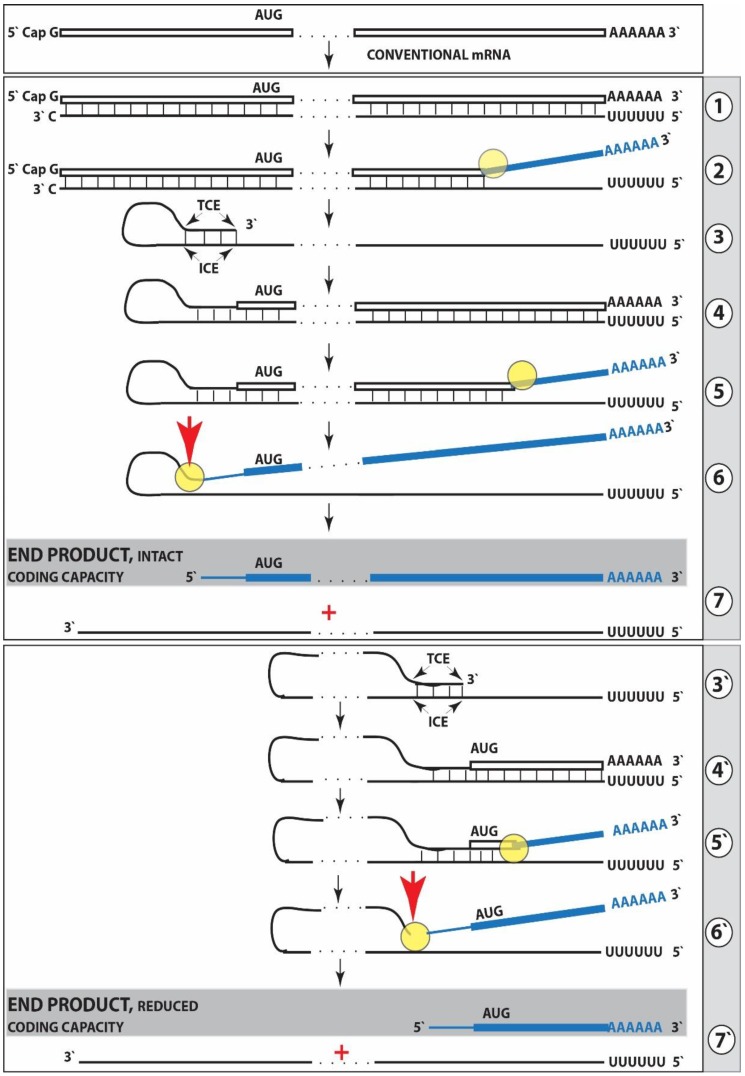
RdRp-mediated amplification of mRNA can result in a 5′-truncated molecule encoding C-terminal fragment of a conventional polypeptide. Boxed line—sense strand RNA. Single line—antisense strand RNA. “AUG”—functional translation initiation codon. “TCE”—3′-terminal complementary element; “ICE”—internal complementary element, both on the antisense strand. Yellow circle—helicase/modifying activity complex. Blue lines (both single and boxed)—RNA strand modified and separated from its complement by a helicase complex. Red arrowhead—position of cleavage of the chimeric intermediate. Top panel—conventional genome-transcribed mRNA molecule. Middle panel—“AUG” is the translation initiation codon of a conventional mRNA and “ICE” of the antisense strand is located within its segment corresponding to the 5′UTR. Chimeric RNA end product contains an intact coding region of a conventional mRNA progenitor and can be translated into the original, conventional mRNA-encoded, polypeptide. Bottom panel—“ICE” is located within a segment of antisense RNA corresponding to the coding region of mRNA. The amplified RNA contains a 5′-truncated coding region of the conventional mRNA progenitor. The translational outcome is decided by the position of the first functional AUG; if in-frame, it initiates translation of a C-terminal fragment of the conventional polypeptide. Steps 3′–7′ correspond to steps 3–7 of the middle panel.

The feasibility of the generation of such 5′-truncated βAPP mRNA forms the second foundation for the proposed mechanism of βAPP-independent production of Aβ and is suggested by the studies of mRNA amplification in other mammalian systems [39,40]. The mechanism for such a process is diagrammed in Figure 1, Steps one through seven. The process of mRNA amplification starts with the antisense complement being transcribed from an mRNA template (Figure 1, Step 1). The generation of a complete antisense transcript requires the presence of an eligible RNA template and a compatible polymerase activity. The only major prerequisite for a potential eligible RNA template appears to be the presence of the poly(A) segment at its 3′ terminus [39,40,41]. The compatible polymerase activity is the RNA-dependent RNA polymerase, RdRp. However, on its own, this, apparently an “omnipresent” core [40], activity has low processivity and terminates after generating only short transcripts [41]. It appears that another activity, a “processivity co-factor” [40] that is induced in selective circumstances, is needed in addition to the core enzyme to efficiently produce the full-length transcripts. A complete antisense transcript of mRNA appears to include, at its 3′ terminus, a “C”, the complement of and a transcript from the 5′-terminal cap “G” of a conventional mRNA molecule (ref. [40]; Figure 1, Steps 1, 2). The resulting double stranded sense/antisense structure is then separated into single-stranded molecules by a helicase activity that mounts the poly(A) segment of the 3′poly(A)-containing strand (the sense-orientation strand) of the double helical structure and proceeds along this strand modifying, on average, every fifth nucleotide in the process (ref. [40]; Figure 1. Step 2). The 5′ poly(U)-containing antisense strand remains unmodified during and after the separation, this being essential for the production of a new sense strand since modifications would interfere with the complementary interactions required in this process and described below.

The vast majority of mammalian mRNA species contains 3′-terminal poly(A) segments. The notion that many, or possibly most, of them could be eligible templates for RdRp was suggested in our previous studies [39]. Subsequent observations by Kapranov and co-workers that showed a widespread synthesis of antisense RNA initiating, apparently indiscriminately, at the 3′ poly(A) of mRNA in human cells [41] expended and generalized this notion. This, apparently undiscerning, RdRp template eligibility of the bulk of mammalian mRNA species raises questions with regard to mechanisms underlying the manifestly stringent specificity of mRNA amplification process as seen, for example, in erythropoietic differentiation [39,40]. The specificity of mRNA amplification process appears to be determined at the 3′ terminus of an antisense transcript by its ability or inability to support the production of a complementary sense strand molecule.

The generation of a sense strand molecule on an antisense template occurs via the extension of the 3′ terminus of a self-primed antisense template and requires the presence within the antisense transcript of two spatially independent complementary elements. One of these is the strictly 3′-Terminal Complementary Element (TCE), the other is the Internal Complementary Element (ICE). These elements (marked, Figure 1, Step 3) must be complementary to a sufficient extent to form a priming structure but may contain mismatches and utilize unconventional G/U pairings. The generation of a sense strand also requires the thermodynamic feasibility, enhanced/enabled by the occurrence of two complementary and topologically compatible elements, of the antisense strand folding into a self-priming configuration. 

Provided that a self-priming structure is formed, the 3′ end of the folded antisense strand is extended by RdRp into a sense-orientation molecule terminating with the poly(A) at the 3′end (Figure 1, Step 4) thus generating a pinhead-structured chimeric intermediate consisting of covalently joined sense and antisense strands. The double stranded portion of the resulting structure is separated by a helicase activity invoked above, which mounts the 3′poly(A) of a newly synthesized sense strand component of the chimeric intermediate and proceeds along this strand in the 5′ direction modifying the molecule as it advances (Figure 1, Step 5). When the helicase activity reaches a single stranded portion of the pinhead structure, it, or the associated activities, cleave the molecule either within the TCE, at a TCE/ICE mismatch, or immediately upstream of the TCE; the cleavage occurs between the 5′ hydroxyl group and the 3′ phosphate (ref. [40]; red arrowhead, Figure 1, Step 6).

Strand separation, in conjunction with the cleavage, produces two single-stranded molecules (Figure 1, Step 7) one of which is a chimeric mRNA, the functional mRNA end product of amplification and the basis for defining this pathway as the “chimeric”. The chimeric nature of this end product is due to the presence at its 5′ end of a 3′-terminal segment of the antisense strand consisting, depending on the site of cleavage of the chimeric intermediate, of either the entire TCE or a portion thereof covalently attached, in a 5′ to 3′ orientation, to the 5′-truncated sense strand. This chimeric molecule is modified and 3′ polyadenylated; it cannot be further amplified because its antisense complement would be lacking the TCE. The extent of 5′-truncation of the sense strand component of chimeric end product is defined by the intramolecular position of the internal complementary element, ICE, within the antisense template. Steps 1 through 7 of Figure 1 depict the situation whereby the ICE of the antisense strand is located within its segment corresponding to the 5′ untranslated region, UTR, of a conventional progenitor mRNA. Consequently, the chimeric end product contains the entire protein coding region of a conventional mRNA and can be translated into the original, conventional mRNA-encoded, polypeptide [40]. 

**Figure 2:**
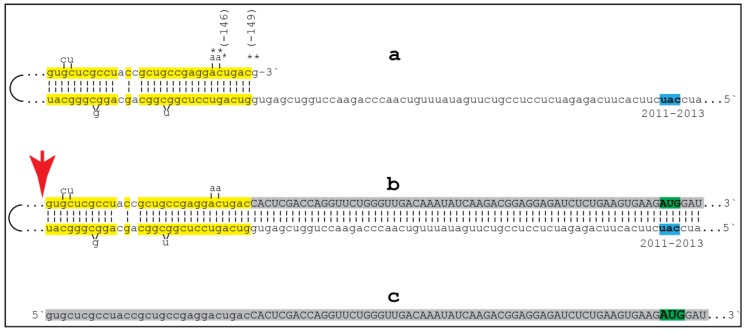
Projected topology of RdRp-mediated generation of 5′-truncated human βAPP mRNA encoding C99 fragment of βAPP. Lowercase letters—nucleotide sequence of the antisense strand. Uppercase letters—nucleotide sequence of the sense RNA strand. Yellow boxes—TCE (top) and ICE (bottom) elements of the antisense strand. Asterisks, (−146) and (−149)—positions on the antisense strand corresponding to transcriptional start sites (TSSs) of human βAPP mRNA (numbering from the AUG translation initiation codon of conventional mRNA). “2011–2013”—positions on the antisense strand of the “uac” (highlighted in turquoise) corresponding to the “AUG” (highlighted in green) encoding Met671 (isoform 770 numbering) in the conventional βAPP mRNA. (a)—TCE/ICE-guided folding of the antisense strand of βAPP mRNA. Note that complementary elements are separated by nearly 2000 nucleotides. (b)—Extension of self-primed antisense strand into sense-orientation RNA and cleavage (after strands-separation) of chimeric intermediate. Note the “AUG” codon (highlighted in green and encoding Met671 in the conventional APP mRNA) 58 nucleotides downstream from the TCE. (c)—Chimeric end product contains, at its 5′ terminus, an antisense sequence (TCE, shown in lowercase letters) extending into severely 5′-truncated βAPP mRNA (shown in uppercase letters). Its translation is initiated from the AUG (highlighted in green) encoding Met 671 (isoform 770 numbering) in the conventional mRNA and it encodes C99 fragment of βAPP.

Potentially, the ICE can be positioned within a segment of the antisense strand corresponding to the coding portion of an mRNA, a scenario diagrammed in steps 3′ trough 7′ of Figure 1. In this scenario, the end product would contain a 5′-truncated coding region of the conventional mRNA progenitor. In such a case, the translational outcome would be decided by the position of the first functional (capable of initiation of translation) AUG. If it is in-frame, translation would result in the C-terminal fragment of a conventional polypeptide (other outcomes are also possible but not relevant for the purposes of the present discussion). To determine if an mRNA species of interest can potentially be amplified by such a mechanism, one needs to assess whether its antisense complement contains both TCE and ICE and is capable of folding into a self-priming configuration. If it is, the position of the ICE would indicate the possible translational outcome. Such an assessment can be conducted in a model experiment where an mRNA of interest serves as a template for synthesis of cDNA (the antisense strand) initiated at the 3′-poly(A) and is subsequently removed by RNAse H activity present in a preparation of reverse transcriptase used. If an mRNA were fully transcribed, if complementary elements were present within the antisense strand, if one of them were 3′-terminal, and if they were topologically compatible, self-priming and the extension synthesis of a segment of the sense strand would occur. The junction between the antisense and sense components would define the site of self-priming and facilitate identification of the TCE and ICE. Just such an experiment was inadvertently carried out with human βAPP mRNA [42]. The results of this experiment, misinterpreted and eventually dismissed by the authors as an artifact [43], indicated the occurrence of topologically compatible TCE and ICE within the antisense strand of βAPP mRNA and defined their sequence as well as the position of self-priming. Based on these results, the TCE/ICE-guided folding of the antisense strand of βAPP mRNA can be diagrammed as shown in Figure 2.

Approximately 30 nucleotide-long 3′-terminal segment of the antisense strand of βAPP mRNA constitutes the TCE. Its counterpart, the ICE, is separated from the TCE by nearly 2000 nucleotides. Yet these elements are topologically compatible and the folding of the antisense molecule results in a self-priming configuration (Figure 2a). The TCE serves as a primer and is extended thus generating the sense strand as shown in the Figure 2b. Strands are then separated as illustrated in Steps 5′–6′ of the Figure 1, and cleavage occurs either at the mismatches within the TCE or immediately upstream from it as indicated by the arrow in Figure 2b and shown diagrammatically in Figure 1, Step 6′. The resulting end-product, diagrammed in Figure 2c, consists of an antisense segment (TCE or its portion) continued into a sense-orientation molecule. The translational outcome is decided by the first, 5′-most, AUG codon; its location and positioning within the sequence of the truncated sense strand is the key for the functionality of the resulting RNA molecule. As can be seen in Figure 2b and c, the first AUG codon is located 58 nucleotides downstream from the TCE portion of the end-product (and 2010 nucleotides from the conventional translation initiation codon of βAPP mRNA if it were an intact molecule), and it is, in fact, the AUG encoding Met671 in the intact βAPP mRNA! The translation, therefore, will result in the 12 kDa C-terminal fragment of the βAPP, that is not susceptible to the α secretase cleavage, and that would become, after the removal of the methionine by the N-terminal methionine aminopeptidase, the C99 which, in turn, after γ-secretase cleavage, will yield the Aβ, an outcome described above in the discussion of an unconventional internal initiation of translation of conventional APP mRNA from the AUG encoding Met671, as was proposed by Breimer and Danny [37], but obtained here by a conventional translation of unconventionally produced mRNA. 

The βAPP gene is in a category of TATA-less genes. In this category, in contrast to TATA-containing genes where the location of the transcription start site, TSS, is strictly controlled by its distance from the TATA element, the position of the TSS is more loosely regulated, and transcription can usually initiate at multiple sites. There are at least five documented TSSs of βAPP mRNA in the normal human brain [44]. The corresponding positions on the antisense strand are marked by asterisks in Figure 2a. In normal subjects, the predominant TSS is at residue -146 (relative to the AUG translation initiation codon). However, only one of these five TSS positions, namely -149, would allow an effective self-priming by the antisense strand and would accommodate in the folding configuration an additional 3′-terminal “C” transcribed from the cap “G”, with other positions being either non-terminal or forming unstable structures. It can be suggested, therefore, that in the development of the SAD the proportion of βAPP transcripts initiating at position -149 increases. Such a shift in the utilization of TSSs can be due to changes in the epigenome of the affected individuals such as histone modifications. Thus, H4K16ac, a key histone modification that is associated with TSSs and regulates cellular responses to stress, is enriched in normal aging, whereas sporadic AD entails dramatic losses of H4K16ac in the proximity of genes linked to aging and AD [33]. Another requirement for the described process to occur is an induction of RdRp, or, rather, of its processivity co-factor. This might occur in response to some cellular stresses and/or epigenetic changes associated with the SAD. It is also conceivable that certain epigenetic changes alone might increase the processivity of the “core” RdRp activity, abrogating the need for a co-factor. Therefore, modulation of βAPP TSS or of RdRp activity could potentially constitute SAD-specific therapeutic approaches.

## 7. Results of Clinical Trials Confirm Key Predictions for Amyloid Precursor Protein-Independent Generation of Beta Amyloid in Sporadic Alzheimer’s Disease

The central prediction of the above mechanism, namely that BACE inhibition should be highly effective in suppressing Aβ production in FAD but would have little effect in SAD, could not be addressed experimentally because animal models of SAD were not and still are not available. Therefore, the best and for now the only approach to test it is in large, statistically significant clinical trials of SAD patients. Such trials, with thousands of participants of different symptomatic levels were just carried out and the result is unequivocal: BACE inhibitor(s), shown safe and effective in healthy subjects and FAD models, had no effect in SAD. These outcomes constitute a confirmation of the key predictions for βAPP-independent production of Aβ in SAD.

It should be emphasized that the notion of the “second component” of Aβ generation in sporadic AD does not challenge the presumption of amyloid hypothesis that SAD is mainly driven by the increased production of Aβ, just as FAD (or rather its subset associated with increased β secretase cleavage) is. It posits, however, that the mechanism of the Aβ production in SAD, or, more specifically, its major component, is different from that in FAD and independent from both βAPP and the activity of β secretase. Conceptually, it introduces two pathways (“components”) of Aβ production. One, which occurs in all systems—healthy subjects, FAD, and SAD—is the excision of Aβ from βAPP requiring cleavages by β and γ secretases; this pathway can be suppressed by β secretase inhibition. Another pathway, that occurs only in SAD, generates, as its primary protein product, C99, which is subsequently cleaved by γ secretase, yielding Aβ; this pathway is resistant to β secretase inhibition. Considering such dynamics of Aβ production, it could be stated that the difference between FAD and healthy subjects is quantitative; i.e., different rates of βAPP cleavage at the β site and different relative rates of cleavage at γ sites—whereas the difference between SAD and FAD (and healthy subjects) is qualitative. 

Conceivably, some cases of SAD can be due to epigenetically mediated changes, for example an increase in β secretase expression in response to the loss of certain microRNA species [45] or the reduction of levels of the potentially neuroprotective peptidase M20-domain-containing protein 1 [46], in the first, βAPP-based, pathway alone. Increase in βAPP gene expression or epigenetic changes resulting in the reduced efficiency of α secretase or of amyloid plaques clearance or in increased use of Aβ position 42 as a γ secretase cleavage site may also elevate the production and/or accumulation of Aβ without the contribution of the second pathway and could result in SAD. Such cases should respond to β secretase inhibition. However, considering the inefficiency of BACE inhibitors in clinical trials, such cases appear to be in the minority. A number of factors must be taken into account when considering the validation of this or any other hypothesis of SAD origin. Thus, age-related blood-brain barrier dysfunction may hinder drug delivery in old SAD patients but not in much younger FAD patients [47]. Additional age-related factors to be considered include increased endoplasmic reticulum (ER) stress and protein folding disturbances, accumulation of mitochondrial mutations, impaired energy generation, and dysfunction of both ubiquitin-proteasome and lysosomal degradation systems.

## 8. BACE Inhibitors Deemed Failed in Sporadic AD Trials Could Be Perfectly Suitable for Treatment of Familial AD

The notion of the “second component” of Aβ production in SAD but not in FAD suggests a decisive and crucial yet feasible and verifiable prediction, namely effective and safe BACE inhibitors would suppress production of Aβ and provide significant benefits, and possibly constitute a “treatment”, in familial AD. If proven, even if it could not be applied to the AD in general, it could provide relief to the quarter of a million current FAD mutation carriers and their descendants in the USA alone. To implement verification of this prediction, an effective and safe BACE inhibitor should be tested with a cohort of FAD patients or descendants of FAD mutations carriers. In fact, if trials of BACE inhibitors that have been concluded or are being evaluated included FAD patients, and if the data for these patients can be extracted and analyzed separately, the trend could become evident. There are currently two FAD-centered trials in progress, DIAN (dominantly inherited Alzheimer network) NCT01760005, and one with the extended Colombian family of FAD carriers aimed at the prevention of the disease, but in both only the immunotherapy approaches are being tested. 

By the same logic, addressing the aspects of AD other than cleavages of the βAPP by β and α secretases should have similar impacts in both FAD and SAD (activation of α secretase could potentially be effective in FAD but not in SAD because the primary product of the second pathway, C99, is not susceptible to α secretase cleavage). Thus, immunotherapy targeting Aβ, or modulation of γ secretase activity toward production of less harmful Aβ species would both be independent of the nature of C99 generation and each approach should be equally effective in FAD and SAD. 

## 9. Lessons from the Trials

The results of the BACE inhibitor trials, thus far persistently negative, should not be dismissed; they contain an important message. While they are being interpreted by some as a verdict on the validity of the amyloid cascade hypothesis, such a judgment appears to be premature; the ACH remains the more versatile putative theory when compared with alternative interpretations of AD. In the framework of the “second component” model for Aβ overproduction in SAD, the lessons from the trials can be formulated as follows: (a) Familial Alzheimer’s disease and sporadic Alzheimer’s disease should be considered two distinctly different diseases as far as the mechanisms of β amyloid generation are concerned. (b) Human trials of BACE inhibitors should be conducted separately, with discrete familial AD and sporadic AD cohorts. (c) It could be expected that BACE inhibitors effective in healthy individuals and in animal studies would also be effective in treatment of FAD. (d) In sporadic AD, two components of Aβ production may be in operation and a substantive portion of Aβ may be generated in βAPP/β secretase cleavage-independent, BACE inhibition-insensitive manner. Therefore, BACE inhibitors would be neither applicable nor effective as a treatment of sporadic AD. (e) Mechanisms of βAPP-independent generation of Aβ in SAD should be further studied; their elucidation could offer SAD-specific therapeutic approaches in addition to modulation of βAPP TSS. (f) Targeting the aspects of Alzheimer’s disease other than β secretase (inhibition) and α secretase (activation) cleavages of the β amyloid precursor protein would have analogous impacts in familial and sporadic AD cases.

## References

[1] Haass C., Lemere C., Capell A., Citron M., Seubert P., Schenk D., Lannfelt L., Selkoe D. (1995). The Swedish mutation causes early-onset Alzheimer’s disease by beta-secretase cleavage within the secretory pathway. Nat. Med..

[2] Dyrks T., Dyrks E., Monning U., Urmoneit B., Turner J., Beyreuther K. (1993). Generation of βA4 from the amyloid protein precursor and fragments thereof. FEBS Lett..

[3] Iizuka T., Shoji M., Kawarabayashi T., Sato M., Kobayashi T., Tada N., Kasai K., Matsubara E., Watanabe M., Tomidokoro Y. (1996). Intracellular generation of amyloid β-protein from amyloid β-protein precursor fragment by direct cleavage with β- and γ-secretase. Biochem. Biophys. Res. Commun..

[4] DeStrooper B., Annaert W. (2000). Proteolytic processing and cell biological functions of the amyloid precursor protein. J. Cell Sci..

[5] Barber R. (2012). The genetics of Alzheimer’s disease. Scientifica.

[6] Vassar R. (2014). BACE1 inhibitors drugs in clinical trials for Alzheimer’s disease. Alzheimer’s Res. Ther..

[7] Beyreuther K., Masters C. (1991). Amyloid precursor protein (APP) and BZA4 amyloid in the etiology of Alzheimer’s disease: Precursor-product relationships in the derangement of neuronal function. Brain Pathol..

[8] Hardy J., Allsop D. (1991). Amyloid deposition as the central event in the aetiology of Alzheimer’s disease. Trends Pharmacol..

[9] Selkoe D. (1991). The molecular pathology of Alzheimer’s disease. Neuron.

[10] Hardy J., Higgins G. (1992). Alzheimer’s disease: The amyloid cascade hypothesis. Science.

[11] Hardy J., Selkoe D. (2002). The amyloid hypothesis of Alzheimer’s disease: Progress and problems on the road to therapeutics. Science.

[12] Selkoe D., Hardy J. (2016). The amyloid hypothesis of Alzheimer’s disease at 25 years. EMBO Mol. Med..

[13] Swerdlow R. (2018). Mitochondria and mitochondrial cascades in Alzheimer’s disease. J. Alzheimer's Dis..

[14] Hunter S., Brayne C. (2018). Understanding the roles of mutations in the amyloid precursor protein in Alzheimer disease. Mol. Psych..

[15] Zhang S., Wang Z., Cai F., Wu Y., Zhang J., Song W. (2017). BACE1 Cleavage Site Selection Critical for Amyloidogenesis and Alzheimer’s Pathogenesis. J. Neurosci..

[16] Vassar R., Bennett B.D., Babu-Khan S., Kahn S., Mendiaz E.A., Denis P., Teplow D.B., Ross S., Amarante P., Loeloff R. (1999). Beta-secretase cleavage of Alheimer’s amyloid precursor protein by the transmembrane aspartic protease BACE. Science.

[17] Hussain I., Powell D., Howlett D.R., Tew D.G., Meek T.D., Chapman C., Gloger I.S., Murphy K.E., Southan C.D., Ryan D.M. (1999). Identification of novel aspartic protease (Asp2) as beta secretase. Mol. Cell. Neurosci..

[18] Sinha S., Anderson J., Barbour R., Basl G., Caccavello R., Davis D., Doan M., Dovey H., Frigon N., Hong J. (1999). Purification and cloning of amyloid precursor protein β-secretase from human brain. Nature.

[19] Wang Y., Strickland C., Voigt J., Kennedy M., Beyer B., Senior M., Smith E., Nechuta T., Madison V., Czarniecki M. (2010). Application of fragment-based NMR screening, X-ray crystallography, structure-based design, and focused chemical library design to identify novel μM leads for the development of nM BACE-1 inhibitors. J. Med. Chem..

[20] Zhu Z., Sun Z., Ye Y., Voigt J., Strickland C., Smith E., Cumming J., Wang L., Wong J., Wang Y. (2010). Discovery of cyclic acylguanidines as highly potent and selective β-site amyloid cleaving enzyme (BACE) inhibitors: Part I—Inhibitor design and validation. J. Med. Chem..

[21] Cumming J., Smith E., Wang L., Misiaszek J., Durkin J., Pan J., Iserloh U., Wu Y., Zhu Z., Strickland C. (2012). Structure based design of iminohydantoin BACE1 inhibitors: Identification of an orally available, centrally active BACE1 inhibitor. Bioorg. Med. Chem. Lett..

[22] Edwards P., Albert J., Sylvester M., Aharony D., Andisik D., Callaghan O., Campbell J., Carr R., Chessari G., Congreve M. (2007). Application of fragment-based lead generation to the discovery of novel, cyclic amidine β-secretase inhibitors with nanomolar potency, cellular activity, and high ligand efficiency. J. Med. Chem..

[23] Barrow J., Stauffer S., Rittle K., Ngo P., Yang Z., Selnick H., Graham S., Munshi S., McGaughey G., Holloway M. (2008). Discovery and X-ray crystallographic analysis of a S-piropiperidine iminohydantoin inhibitor of β-secretase. J. Med. Chem..

[24] Malamas M., Erdei J., Gunawan I., Turner J., Hu Y., Wagner E., Fan K., Chopra R., Olland A., Bard J. (2010). Design and synthesis of 5,5′-disubstituted aminohydantoins as potent and selective human β-secretase (BACE1) inhibitors. J. Med. Chem..

[25] Rueeger H., Rondeau J., McCarthy C., Moebitz H., Tintelnot-Blomley M., Neumann U., Desrayaud S. (2011). Structure based design, synthesis and SAR of cyclic hydroxyethylamine (HEA) BACE-1 inhibitors. Bioorg. Med. Chem. Lett..

[26] Probst G., Xu Y. (2012). Small-Molecule BACE1 Inhibitors: A patent literature review (2006−2011). Expert Opin. Ther. Patents.

[27] May P., Dean R., Lowe S., Martenyi F., Sheehan S., Boggs L., Monk S., Mathes B., Mergott D., Watson B. (2011). Robust central reduction of amyloid-β in humans with an orally available, non-peptidic β-secretase inhibitor. J. Neurosci..

[28] Stamford A.W., Scott J.D., Li S.W., Babu S., Tadesse D., Hunter R., Wu Y., Misiaszek J., Cumming J.N., Gilbert E.J. (2012). Discovery of an orally available, brain penetrant BACE1 inhibitor that affords robust CNS Aβ reduction. ACS Med. Chem. Lett..

[29] Kennedy M.E., Stamford A.W., Chen X., Cox K., Cumming J.N., Dockendorf M.F., Egan M., Ereshefsky M., Hodgson R.A., Hyde L.A. (2016). The BACE1 inhibitor verubecestat (MK-8931) reduces CNS b-amyloid in animal models and in Alzheimer’s disease patients. Sci. Transl. Med..

[30] Keskin A.D., Kekuš M., Adelsberger H., Neumann U., Shimshek D.R., Song B., Zott B., Peng T., Förstl H., Staufenbiel M. (2017). BACE inhibition-dependent repair of Alzheimer’s pathophysiology. Proc. Natl. Acad. Sci. USA.

[31] Neumann U., Rueeger H., Machauer R., Veenstra S.J., Lueoend R.M., Tintelnot-Blomley M., Laue G., Beltz K., Vogg B., Schmid P. (2015). A novel BACE inhibitor NB-360 shows a superior pharmacological profile and robust reduction of amyloid-β and neuroinflammation in APP transgenic mice. Mol. Neurodegen..

[32] Hu X., Das B., Hou H., He W., Yan R. (2018). BACE1 deletion in the adult mouse reverses preformed amyloid deposition and improves cognitive functions. J. Exp. Med..

[33] Nativio R., Donahue G., Berson A., Lan Y., Amlie-Wolf A., Tuzer F., Toledo J.B., Gosai S.J., Gregory B.D., Torres C. (2018). Dysregulation of the epigenetic landscape of normal aging in Alzheimer’s disease. Nat. Neurosci..

[34] Volloch V. (1996). A mechanism for ß-amyloid overproduction in Alzheimer’s disease: Precursor-independent generation of ß-amyloid via antisense RNA-primed mRNA synthesis. FEBS Lett..

[35] Volloch V., Wasco W., Tanzi R. (1997). Mechanism for β-amyloid overproduction in Alzheimer’s Disease: Possible antisense RNA-mediated generation of a 5’-truncated βAPP mRNA encoding 12 kDa C-terminal fragment of βAPP, the immediate precursor of Aß. Molecular Mechanisms of Dementia.

[36] Volloch V. (1997). Possible mechanism for resistance to Alzheimer's disease (AD) in mice suggests new approach to generate a mouse model for sporadic AD and may explain familial resistance to AD in man. Exp. Neurobiol..

[37] Breimer L., Denny P. (1987). Alzheimer amyloid aspects. Nature.

[38] Citron M., Haass C., Selkoe D. (1993). Production of amyloid-β-peptide by cultured cells: No evidence for internal initiation of translation at Met_596_. Neurobiol. Aging.

[39] Volloch V., Schweitzer B., Rits S. (1996). Antisense globin RNA in mouse erythroid tissues: Structure, origin, and possible function. Proc. Natl. Acad. Sci. USA.

[40] Rits S., Olsen B., Volloch V. (2016). RNA-dependent synthesis of mammalian mRNA: Identification of chimeric intermediate and putative end-product. BioRxiv.

[41] Kapranov P., Ozsolak F., Kim S., Foissac S., Lipson D., Hart C., Roels S., Borel C., Antonarakis S., Monaghan A. (2010). New class of gene-termini-associated human RNAs suggests a novel RNA copying mechanism. Nature.

[42] Mita S., Sadlock J., Herbert J., Schon E. (1988). A cDNA specifying the human amyloid beta precursor protein encodes a 95-kDa polypeptide. Nucl. Acids Res..

[43] Mita S., Sadlock J., Herbert J., Schon E. (1988). A cDNA specifying the human amyloid beta precursor protein encodes a 95-kDa polypeptide: CORRECTION. Nucl. Acids Res..

[44] Salbaum J., Weidemann A., Lemaire H., Masters C., Beyreuther K. (1988). The promoter of Alzheimer’s disease A4 precursor gene. EMBO J..

[45] Hebert S., Horre K., Nicolaï L., Papadopoulou A., Mandemakers W., Silahtaroglu A., Kauppinen S., Delacourte A., De Strooper B. (2008). Loss of microRNA cluster miR-29a/b-1 in sporadic Alzheimer’s disease correlates with increased BACE1/beta-secretase expression. Proc. Natl. Acad. Sci. USA.

[46] Zanchez-Mut J.V., Heyn H., Silva B.A., Dixsaut L., Garcia-Esparcia P., Vidal E., Sayols S., Glauser L., Monteagudo-Sánchez A., Perez-Tur J. (2018). PM20D1 is a quantitative trait locus associated with Alzheimer’s disease. Nat. Med..

[47] Sweeney M., Sagare A., Zlokovic B. (2018). Blood-brain barrier breakdown in Alzheimer disease and other neurodegenerative disorders. Nat. Rev. Neurol..

